# Reimagining and rebranding advance care planning

**DOI:** 10.1093/ageing/afae233

**Published:** 2024-10-24

**Authors:** Chetna Malhotra

**Affiliations:** Duke-NUS Medical School, Lien Centre for Palliative Care, Program in Health Services and Systems Research, 8 College Road Singapore 169857

**Keywords:** advance care planning, care planning, care preparation, end of life, serious illness, goal concordant care, older people

## Abstract

Advance care planning (ACP) has traditionally aimed at ensuring that patients’ end-of-life (EOL) wishes are understood and respected. However, recent literature raises concerns about its effectiveness, with many trials indicating that ACP does not significantly improve goal-concordant care, enhance quality of life or reduce healthcare costs. This is because patients’ future decisions are influenced by their transient preferences due to projection bias. To remain relevant, ACP requires a radical shift in perspective, implementation and branding. First, ACP’s mission must be redefined with a focus on: Educate, Share and Prepare. This perspective emphasises ongoing conversations about patient health and illness, sharing of patients’ current values and goals of care and preparation for the future, rather than making definitive future decisions. Second, ACP should be integrated into routine care, normalising these discussions. Simplifying ACP processes and shifting incentives to support shared responsibility among stakeholders can enhance integration. Last, rebranding ACP as ‘Advance Care Preparation’ can clarify its purpose, distinguishing it from EOL planning and increasing its uptake. This rebranding ensures that ACP meets the evolving needs of patients and their families, ultimately enhancing the quality of care and patient satisfaction. These changes in perspective, implementation and branding can transform ACP into a valuable tool for delivering compassionate, patient-centred healthcare, making it relevant to all individuals.

## Key Points

- Recent literature has raised concerns about the effectiveness of advance care planning (ACP).
- To remain relevant, ACP requires a radical shift in perspective, implementation and branding.
- ACP should focus on a tri-fold mission: Educate, Share and Prepare.
- ACP must be integrated into ongoing care rather than being treated as a standalone task designated solely for the future.
- ACP must be rebranded as ‘Advance Care Preparation’ to help synchronise stakeholders’ mental models of what it is.

Advance care planning (ACP) has long been heralded as a cornerstone of patient-centred care, aiming to ensure that patients’ wishes for end-of-life (EOL) care are understood and respected. However, recent literature has ignited a contentious debate regarding its efficacy and value [1]. A recent systematic review reported that a large number of trials conducted among patients who are not imminently dying patients have failed to show that ACP improves goal-concordant care at the end of life, enhances patient quality of life, or reduces healthcare costs [2]. Evidence of ACP leading to goal-concordant care was found only in studies involving imminently dying patients [2]. Thus, the available evidence does not necessarily support that ACP changes patients’ EOL trajectory.

This implies that the traditional perspective on ACP—with its objective of facilitating goal-concordant care or perhaps reducing healthcare costs at the end of life—no longer meets the needs of modern healthcare systems and the patients they serve. In light of this new knowledge, ACP risks losing its relevance unless it is reimagined and rebranded.

To reimagine ACP, we first need to redefine its mission [3, 4]. The traditional approach to ACP, which focuses on ‘planning for EOL care’, is relevant only for those who are imminently dying because it overlooks a critical reality: patients’ and caregivers’ goals and preferences are transient [5–8], often reflecting their current state of mind rather than what they may want in the future, a phenomenon known as projection bias. As a result, making definitive decisions for future EOL care should not be a goal for ACP. Instead, ACP should focus on a tri-fold mission: Educate, Share and Prepare. Under this tri-fold mission, ACP empowers patients with the knowledge of current health and illness trajectory and facilitates sharing of what truly matters to them right now, driving engagement in shared decision-making for their immediate care. Ongoing illness education and sharing of patient values, along with the appointment of a surrogate decision maker, lay the groundwork for preparing patients and families for difficult future healthcare decisions. This framework acknowledges the inherent uncertainties of planning for the future. Instead, it allows patients to be informed about their health, plan for current care and prepare for future care decisions, thus shifting the emphasis to more immediate and actionable aspects. This approach is relevant for all individuals, including those who are seriously ill, and it establishes a more realistic and pragmatic basis for measuring ACP’s success.

This shift in ACP’s mission requires that all stakeholders, including those funding ACP resources, implementation and research, value ACP’s proposition as a preparatory tool for future care. While some may consider preparation as a proximal or process outcome, it underscores the inherent value of ACP in delivering compassionate and patient-centred healthcare.

Second, ACP must be integrated into ongoing care rather than being treated as a standalone task designated solely for the future. Viewing ACP as something solely for the future leads to procrastination by individuals and patients to engage in ACP and often means that ACP is only addressed in times of emergency when patients and families are already under significant stress and least prepared for these discussions [9]. To rectify this, ACP should be viewed as an integral part of ongoing care, becoming a continuous conversation, evolving alongside the patient’s health status and personal circumstances [3]. This approach normalises ACP conversations rather than being an uncomfortable topic to be avoided, thereby potentially increasing its uptake, especially in cultural contexts where talking about death and dying remains taboo [10].

In order for ACP to be integrated in ongoing care, ACP conversations and forms should be simplified [10]. Currently, the ACP conversations and documentations are time-consuming, and with limited consultation time, clinicians have little room to integrate these in routine care [11]. Simplifying these processes reduces the burden on both patients and healthcare providers, making ACP a more manageable and less daunting task. Further, these conversations should be allowed to evolve over multiple visits without requiring that the entire conversation and form be completed in one visit. Electronic health records should facilitate documentation of these bite-sized ACP conversations.

Integration also requires a significant shift in how ACP is incentivised. In many countries, ACP programmes are implemented by healthcare systems with a strong focus on metrics, such as the number of completed ACP forms [10]. While this structured approach guarantees funding and resources, it misses the essence of ACP—that it should ensure meaningful and individualised care for each patient. It also places the onus of initiating ACP conversations primarily on healthcare providers. Instead, ACP should be a shared responsibility between patients, healthcare professionals, families, society and various institutions. Patient-focused tools such as ACP decision aids can be used to coach and empower patients in taking responsibility of articulating their healthcare goals. Healthcare providers should encourage patients to engage in ACP conversations during routine check-ups or hospital visits. Families can support their loved ones by being active participants in ACP discussions and advocating for them when necessary. Healthcare institutions should create environments and triggers that facilitate ACP conversations during healthcare visits, including integrating ongoing ACP into electronic health records to ensure accessibility and continuity of care.

Last, ACP must be rebranded as ‘Advance Care Preparation’ to help synchronise stakeholders’ mental models of ACP and communicate its new identity and trifold mission of Educate, Share and Prepare. Currently, some clinicians perceive ACP as a tool for achieving goal-concordant care [12]. Policymakers often link ACP with efforts to reduce healthcare costs [13]. For many patients, ACP is synonymous with EOL decision-making. These disparate interpretations underscore the need for a unified understanding of ACP.

While some may argue that the name is inconsequential, they neglect the psychological and social barriers that specific terminology can create. Yet, in healthcare, terminology holds substantial influence. Names are not mere labels; they are powerful communicators of the intent and scope of care that patients and caregivers are receiving [14]. Names also shape perceptions and expectations regarding care. Further, marketing strategies suggest that rebranding can distance entities from previous negative associations, allowing them to communicate new and improved values. Companies like Facebook (now Meta) have rebranded to reflect their evolving missions and goals. Closer home, several palliative care departments rebranded themselves as supportive care departments, placing palliative care more upstream within patient care and reducing stigma around its use [15].

Rebranding ACP can help synchronise stakeholders’ mental models of ACP and communicate its identity as a process for preparing patients and families for future care decisions. It does not give false ideas or promises to patients and caregivers that ACP can help achieve goal-concordant care. Instead, it provides assurance that the process is dynamic and ongoing. It also positions ACP more upstream, potentially increasing its uptake, as patients and caregivers recognise their value throughout the illness trajectory, not just at its terminal stages. Retaining the acronym ACP while rebranding to ‘Advance Care Preparation’ could facilitate a smoother transition and minimise the need for a complete overhaul. Since there is a risk that stakeholders may not fully understand the shift from ‘planning’ to ‘preparation’, careful communication will be essential to effectively convey the change in intent.

To conclude, ACP requires a radical shift in perspective, implementation and branding. By redefining its mission, integrating it into ongoing care and rebranding it to reflect its true purpose, we can transform ACP into an effective tool for the provision of patient-centred care. As a next step, appropriate measures must be developed and validated to evaluate the success of ACP in fulfilling its tri-fold mission. This will ensure a systematic evaluation of whether the shift in ACP genuinely meets the needs of patients and their families and improves their care quality and satisfaction.
